# Persistence of specialized bacteria during disinfectant challenge in a new swimming pool

**DOI:** 10.1016/j.engmic.2026.100261

**Published:** 2026-02-05

**Authors:** Ziyu Wan, Wanzhen Guo, Kaiyue Zhang, Lianfeng Tang, Youming Zhang, Hai Xu, Mengge Zhang, Xueyun Geng, Ling Li, Wenjia Wang, Mingyu Wang

**Affiliations:** a State Key Laboratory of Microbial Technology, Microbial Technology Institute, Shandong University, Qingdao 266237, China; b Taishan College, Shandong University, Jinan 250010, China; c School of Life Sciences, Shandong University, Qingdao 266237, China; d School of Physical Education, Shandong University, Qingdao 266237, China; e Qilu Hospital Qingdao, Cheeloo College of Medicine, Shandong University, Qingdao 266035, China; f Shanghai Key Laboratory of Atmospheric Particle Pollution and Prevention (LAP), Shanghai 200443, China

**Keywords:** Swimming pool, Bacterial community structure, Whole genome sequencing, Disinfectant resistance, Plasmids, Complex class I integron

## Abstract

Swimming pools represent a unique aquatic environment, with continuous bacterial inoculation from swimmers and high disinfectant levels to control microbial growth. Whether, how, and which groups of bacteria can survive this strong disinfectant stress and pose a potential threat to swimmer health remain poorly understood. This study leveraged the unique opportunity of a newly opened swimming pool to address these questions. Analysis of bacterial community structures in water revealed that disinfectants substantially and temporarily simplified bacterial communities, with only a few dominant specialized groups surviving under disinfectant stress. Among the culturable pathogens, such as *Escherichia coli, Staphylococcus*, and *Acinetobacter*, only specific strains possessing genetic adaptations, such as stress-tolerant plasmids, persisted. Although most of the isolated strains did not exhibit high levels of antibiotic resistance, the presence of resistant pathogens was confirmed, one of which carried a unique complex Class I integron. This study demonstrates how bacteria emerge and persist in heavily managed, disinfected aquatic environments, such as swimming pools. Consequently, there is an urgent need to shift focus from merely reducing bacterial populations to developing sustainable strategies for managing persistent bacteria to protect public health.

## Highlights

• Disinfection significantly and temporarily simplified bacterial community in a new swimming pool.
• Environmental bacteria possessing specialized metabolic capabilities or harboring specialized plasmids can persist under disinfectant exposure.
• A new complex Class I integron was found in *Acinetobacter lwoffii*.

## Graphical abstract

**Figure:**
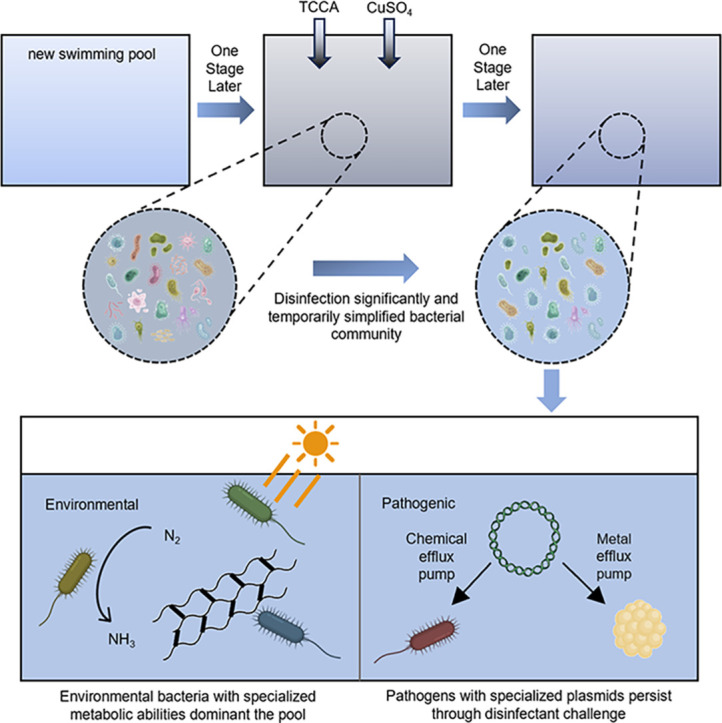

## 1 Introduction

Swimming pools are one of the most common water bodies shared by people. In most cases, it is accessible to non-specific individuals with limited restrictions. It also has a high population density compared to other water bodies. Therefore, swimming pools can have a high microbial content, making them a potential pathway for transmitting infectious diseases. A growing body of evidence from diverse geographical regions has documented that recreational water bodies, particularly swimming pools, are associated with an elevated risk of bacterial infection [[1], [2], [3]]. To address this issue, high concentrations of disinfectants are routinely added to swimming pools, which generally perform well in killing pathogens in swimming pool water. Nevertheless, pathogens, such as *Escherichia coli, Pseudomonas aeruginosa*, and *Cryptosporidium* can still be detected in swimming pools [4,5]. Moreover, there have been reports of methicillin-resistant *Staphylococcus aureus* strains isolated from swimming pool water [6].

Advancements in sequencing technologies have facilitated comprehensive research on swimming pool microbiota. Although traditional culture-based methods are effective in detecting cultivable pathogens, they provide limited insights into the overall microbial community structure and miss unculturable microorganisms [7]. To overcome these limitations, high-throughput sequencing, including 16S rRNA amplicon sequencing, enables the comprehensive profiling of microbial composition and community structure in water samples [7,8]. However, this approach alone does not reveal the genetic determinants of the individual strains. Whole-genome sequencing (WGS) addresses this gap by allowing the precise characterization of genetic features, such as stress-response genes, virulence factors, and antibiotic resistance genes (ARGs), which underlie microbial persistence in challenging environments [9]. Thus, the integration of high-throughput sequencing with WGS offers a powerful strategy for elucidating the composition and functional attributes of bacterial populations in environmental settings. However, such studies have rarely been conducted using swimming pool water. Among a limited number of reports, Ma et al. described the metagenomic and resistomic changes in microbes in laboratory-scale wastewater studies before and after chlorination, mimicking the concentration used in swimming pools and drinking water [10], focusing on changes in ARGs. Other related environments such as children’s paddling pools have also been studied [11]. These studies have primarily relied on high-throughput metagenomic sequencing to investigate microbial population dynamics. While the aforementioned studies primarily focused on global shifts in microbial populations, the behavior and characteristics of individual bacteria in swimming pools remain largely overlooked.

This work attempted to carry out an extensive study on microbes in a full-scale, well-operated swimming pool, taking advantage of modern sequencing technologies and taking the opportunity to open a new swimming pool at the Qingdao Campus, Shandong University. It has rarely been observed how microbes change from a pristine, unused swimming pool to a pool affected by continuous human activity. This is one of the first studies to determine whether and how certain bacterial groups survive the strong disinfectant stress present in swimming pools. Persistent bacteria and their genetic determinants can function as novel microbial indicators for environmental monitoring, analogous to the human-specific biomarker coprostanol used for data normalization in wastewater surveillance [12]. These indicators are critical for assessing the gap in disinfection efficacy and potential health risks in swimming pools. Therefore, monitoring these specific persistent bacteria rather than relying solely on total bacterial counts could provide a more precise and efficient early warning strategy for water safety. As this study was conducted in a well-operated pool with strict disinfection protocols, it demonstrates that a potential infection threat can persist even in environments deemed safe, and introduces a novel concept for environmental monitoring.

## 2 Materials and methods

### 2.1 Sample collection and bacterial isolation

Samples were collected from the swimming pool of the gymnasium at Shandong University, Qingdao Campus. Between January 10th and April 13th, 2024, 12 water samples were collected weekly. Sample collection nearly reached the full operational cycle of the pool, defined as the first sample collected on the opening day before swimmer use, while the final sample was collected immediately prior to routine drainage. Swimming pool water sampling and bacterial collection were performed as previously described [13].

All samples were collected in sterile sealed plastic buckets with a total capacity of approximately 18.4 liters. The samples were collected at a point approximately 10 cm away from the pool edge and a depth of 10 cm and then transferred to the laboratory for further analysis. The water samples were filtered using a sand core filter (1000 mL) and a microporous membrane with a pore size of 0.22 μm. Microporous membranes containing the filtered bacteria were cut into small pieces and immersed in 0.01 M phosphate-buffered saline (pH 7.2) for approximately 2.5 h at room temperature (20–25 °C). After vortexing for 10 min, 200 μL of the supernatant was plated on Luria-Bertani (LB) agar plates and incubated overnight at 37 °C, with the remainder used for subsequent genomic extraction. Individual colonies with different morphologies were selected for subsequent experiments.

### 2.2 Genome extraction and sequencing

The genomic DNA of the isolated bacterial strains was extracted using a TIANamp Bacteria DNA Kit (TIANGEN BIOTECH, Beijing, China). A Qubit dsDNA HS Assay Kit (Invitrogen, Waltham, MA, USA) was used to quantify the genomic DNA concentration. A Rapid Barcoding Kit 96 V14 (SQK-RBK114.96, Oxford Nanopore Technologies, Oxford, UK) was used to construct a DNA library. A Nanopore P2solo sequencer (Oxford Nanopore Technologies, Oxford, UK) was used for genome sequencing.

Total genomic DNA was extracted from water samples using a TIANamp Soil DNA Kit (TIANGEN BIOTECH, Beijing, China). Amplicon sequencing of the 16S rDNA V4 region was performed by Novogene Co., Ltd. (Beijing, China). Sequencing was conducted on an Illumina NovaSeq sequencer (Illumina, Inc., San Diego, CA, USA) in PE250 mode.

### 2.3 Filtering, assembly, and annotation of sequencing data

Raw 16S rDNA amplicon sequencing data were analyzed using Quantitative Insights into Microbial Ecology 2 (QIIME 2). Flye v2.9.2-b1786 was used for Nanopore sequence genome assembly [14], and Medaka v1.11.3 (https://github.com/nanoporetech/medaka) was subsequently used for error correction. QUAST v5.0.2 [15], CheckM2 v1.0.2 [16], and BUSCO v5.2.2 [17] were used to evaluate the quality and completeness of the genome assembly. GTDB-Tk v2.1.1 was used for the taxonomic annotation of the assembled genome [18]. Gene prediction and annotation were performed using PGAP v6.7 (build 7555, released July 18, 2024) [19], and Prokka v1.14.6 [20]. ARGs were predicted using AMRfinder v3.11.1, with database version 2023–04–17.1 [21] and RGI (https://card.mcmaster.ca/analyze/rgi), whereas plasmid typing was performed using PlasmidFinder [22]. The plasmids were further confirmed using a BLAST search against the GenBank NT database. The contig circularity and copy number were calculated using the Flye software. Comparison of genomes and identification of mutations was performed with Snippy v4.6.0 (https://github.com/tseemann/snippy) and Gubbins v3.3.1 [23].

### 2.4 Antimicrobial susceptibility tests

Antimicrobial susceptibility tests were performed on isolates of *E. coli, Staphylococcus*, and *Acinetobacter* using the K–B disk diffusion method, following Clinical Laboratory Standards Institute procedures.

### 2.5 Data analysis

Alpha-diversity was computed in QIIME2, and statistical trends across samples were assessed via linear regression in R. A phylogenetic tree was constructed using FastTree [24,25] and visualized with iTOL [26]. Plasmid visualization was performed using SnapGene v8.0.1. Plasmid replicon types were predicted using the PlasmidFinder software. Mobile genetic elements (MGEs) on the plasmids were identified using MOB-suite. The homologous sequences in the database were searched using BLAST. Comparative visualization of plasmid homology was performed using Easyfig. Fig. 5 was created using https://BioRender.com.

### 2.6 Genomic sequence data deposition

All genomic sequence data were deposited in GenBank under the accession number PRJNA1211488. The accession numbers for the individual strains are listed in Table S1.

## 3 Results

### 3.1 Sampling of swimming pool water

Water samples were collected from the swimming pool of Shandong University, Qingdao Campus. This swimming pool was opened on January 10th, 2024, providing a unique opportunity to observe changes in microbial communities in pool water resulting from anthropogenic activities and disinfection, as well as the evolution of pathogenic potential within the swimming pool.

Water samples were collected from the swimming pool in the morning approximately every week. Twelve samples were collected from January 10th to April 13th, 2024. The first sample was taken on January 10th, before the swimming pool was officially opened to the public. This period also included the Spring Festival (Chinese New Year) when students were on vacation and swimming activities were low (Table S1).

**Fig. 1:**
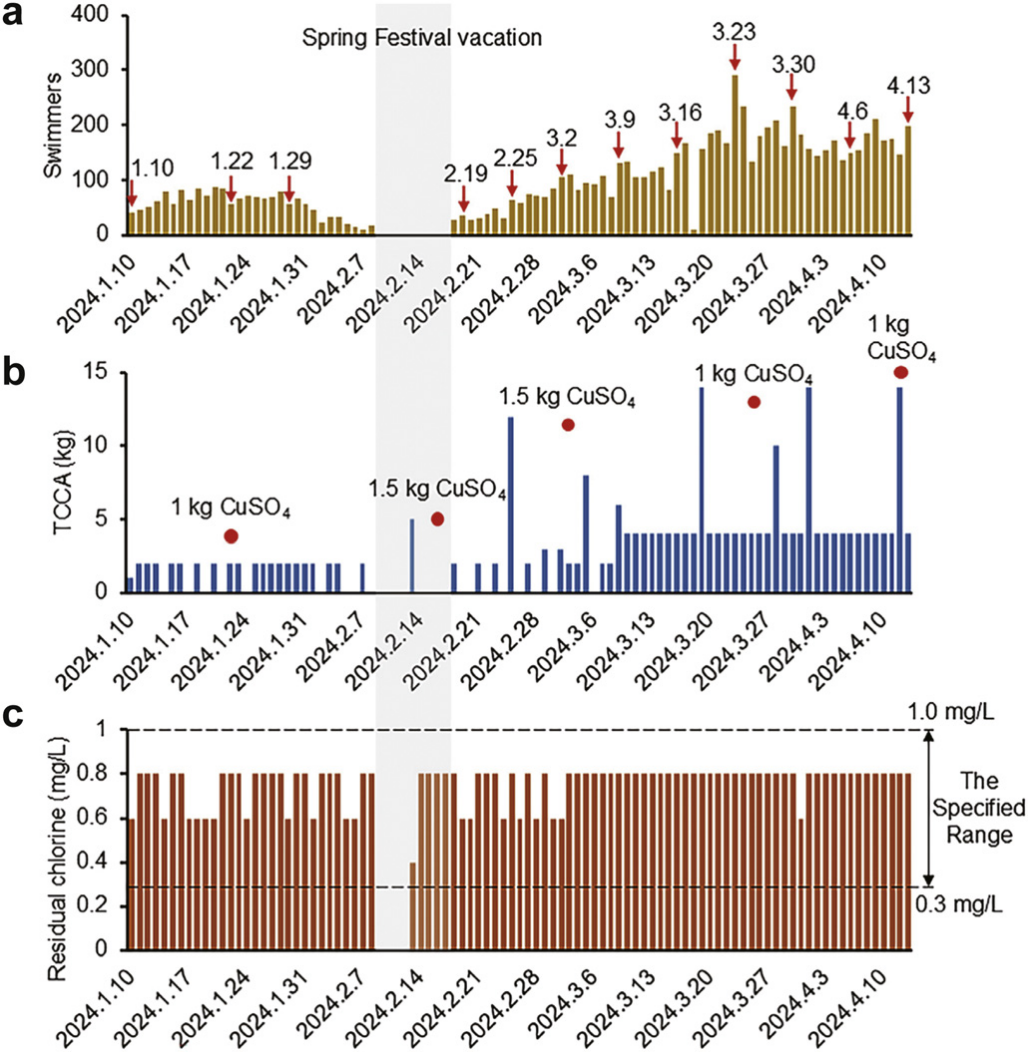
Swimming pool operation statistics. (a) Number of swimmers, (b) addition of disinfectants, (c) residual chlorine. The red arrows indicate sampling dates. The gray area indicates the Spring Festival vacation period when the gymnasium was closed. The addition of CuSO_4_ was noted in (b). TCCA, trichloroisocyanuric acid.

The number of swimmers in the pool showed a general increasing trend since its opening (Fig. 1a). An obvious gap was seen during the Spring Festival vacation when the swimming pool was closed, which led to a ‘break’ in both swimming activities and disinfection. Routine disinfection was performed through the daily addition of trichloroisocyanuric acid (TCCA) (Fig. 1b). CuSO_4_ was also added monthly. Residual chlorine remained at a healthy level between 0.4 and 0.8 mg/L (Fig. 1c), complying with the Chinese standard range of 0.3–1.0 mg/L (National Standards of the People’s Republic of China, GB 37,488–2019), suggesting effective disinfection. In general, this is a well-operated, reasonably populated, and routinely disinfected swimming pool that can be considered representative of a typical swimming pool during opening.

### 3.2 Simplification of bacterial communities and persistence of metabolically specialized groups

**Fig. 2:**
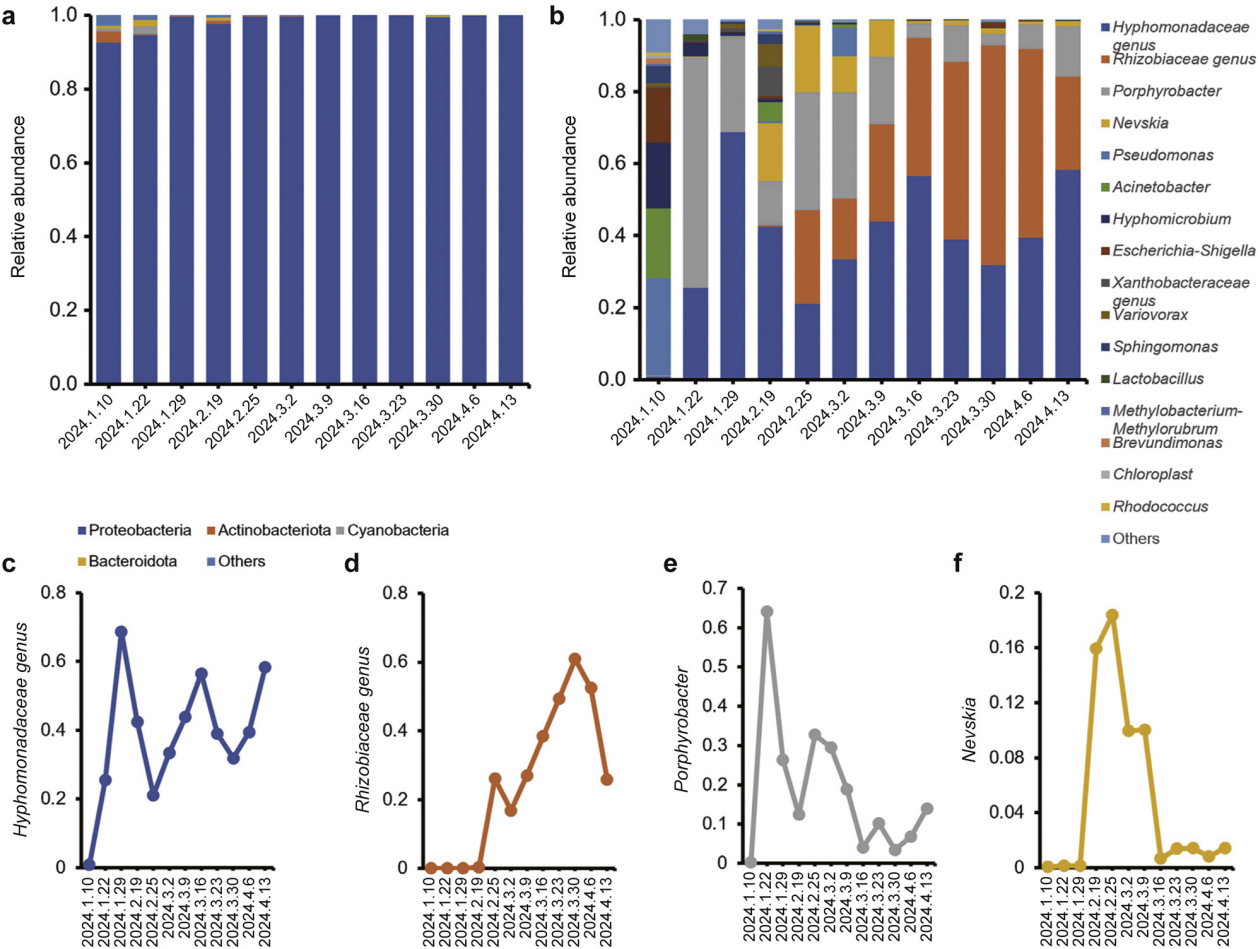
Microbial communities of swimming pool samples. The results are shown at the (a) phylum level, (b) genus level, (c) Hyphomonadaceae family level; (d) Rhizobiaceae family level, (e) *Porphyrobacter* genus level, and (f) *Nevskia* genus level.

The characteristics of the overall bacterial community structure were studied using 16S rDNA amplicon sequencing. In general, Proteobacteria was the dominant phylum (Fig. 2a), although a small fraction of microbes before and soon after the opening of the swimming pool belonged to other phyla.

Quick and effective removal of human-related bacteria was observed in the swimming pool water following the opening of the pool and the application of disinfectants. Prior to the opening of the swimming pool, the pool bacterial community was primarily composed of human-related microbes including *Escherichia-Shigella, Pseudomonas*, and *Acinetobacter*, along with the denitrifying bacteria *Hyphomicrobium* [27] (Fig. 2b). This composition drastically changed following the opening of the swimming pool, with quick, near-complete elimination of human-related bacteria (Fig. 2b), consistent with the purpose of disinfection.

**Fig. 3:**
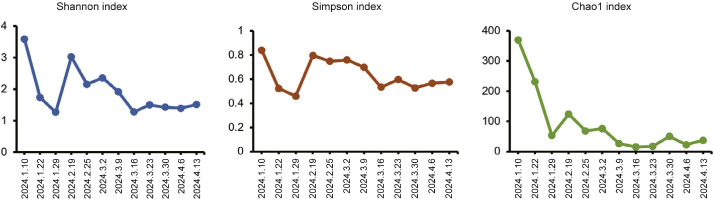
Alpha indices of the samples.

In addition to the removal of human-related pathogenic bacteria, simplification of the bacterial community in pool water was observed. Following the opening of the swimming pool, the α-diversity exhibited a significant overall decline (Shannon index: –0.124, Chao1 index: –22.059 per time unit; both *p* < 0.05). This decline was particularly pronounced during the initial phase preceding the Spring Festival (Fig. 3). However, this simplified bacterial community is temporary and depended on the continuous disinfectant pressure. During the Spring Festival of 2024, the α-diversities were restored due to the reduction of disinfection frequency (Fig. 3), although the human-mediated inoculation of microbes also stopped. These findings suggest that although disinfection can lead to simplified bacterial communities in swimming pools, it is also necessary to maintain this simplified structure.

Examination of swimming pool bacterial community structures revealed the persistence of specific bacterial groups. Generally, four microbes dominated the microbial communities during the operation of the swimming pool: genera of the families Hyphomonadaceae and Rhizobiaceae, as well as the genera *Porphyrobacter* and *Neviskia* (Fig. 2b). Before the Spring Festival, Hyphomonadaceae and *Porphyrobacter* were the dominant taxa, whereas after the festival, all four microbial groups, including Rhizobiaceae and *Nevskia*, were detected. These groups of bacteria persisted despite routine disinfection, showing that even under strong disinfectant stress, the swimming pool could not completely remove microbes (Fig. 2c–2f).

The bacterial groups that persisted in the swimming pool were metabolically specialized. Microbes of the family Hyphomonadaceae are commonly found among marine diazotrophic bacteria [28,29]. Bacteria of the genus *Porphyrobacter* have been implicated in connection with microalgae and cyanobacteria [30,31], and were found to be anoxygenic phototrophic bacteria in seawater [32]. *Nevskia* is commonly found in the soil [33], and has been shown to degrade complex organic substances [34]. Rhizobiaceae are diazotrophic bacteria commonly found in soil [35]. Swimming pools are nutrient-scarce environments in which carbon and nitrogen sources are limited. Therefore, these metabolically specialized bacteria have better fitness than the commonly observed bacteria in nutrient-rich environments.

### 3.3 Persistence of specialized pathogenic bacteria under strong disinfectant stress

A swimming pool is an environment with strong disinfectant stress, especially with the daily application of TCCA and monthly treatment with CuSO_4_. The ability of pathogenic bacteria to survive strong disinfectant stress was studied using a culture approach. A total of 83 bacterial isolates belonging to 17 species and 7 genera were isolated from swimming pool water samples (Table 1). The four most common genera were *Bacillus, Escherichia, Staphylococcus*, and *Acinetobacter*. Although *Bacillus* is commonly found among environmental bacteria, other genera are often found in humans. Forty-nine isolates (59 % of all isolates), including *E. coli*and *Staphylococcus aureus*, are well-known opportunistic pathogens. Whole-genome sequences of 83 bacterial isolates were obtained and annotated, using 3rd generation Nanopore sequencing, which can generate near-perfect genomic assemblies. Plasmids were identified and sequenced using this approach.

**Table 1:** Bacteria isolated in this study.

Species	Isolate number
*Bacillus* (38 strains)	
*Bacillus cereus*	15
*Bacillus velezensis*	15
*Bacillus thuringiensis*	4
*Bacillus licheniformis*	2
*Bacillus safensis*	1
*Bacillus subtilis*	1
*Staphylococcus* (14 strains)	
*Staphylococcus saprophyticus*	5
*Staphylococcus aureus*	4
*Staphylococcus haemolyticus*	2
*Staphylococcus epidermidis*	1
*Staphylococcus hominis*	1
*Staphylococcus lugdunensis*	1
*Acinetobacter* (9 strains)	
*Acinetobacter lwoffii*	9
Others (22 strains)	
*E. coli*	19
*Kocuria palustris*	1
*Neobacillus* sp.*005154805*	1
*Priestia zanthoxyli*	1

**Fig. 4:**
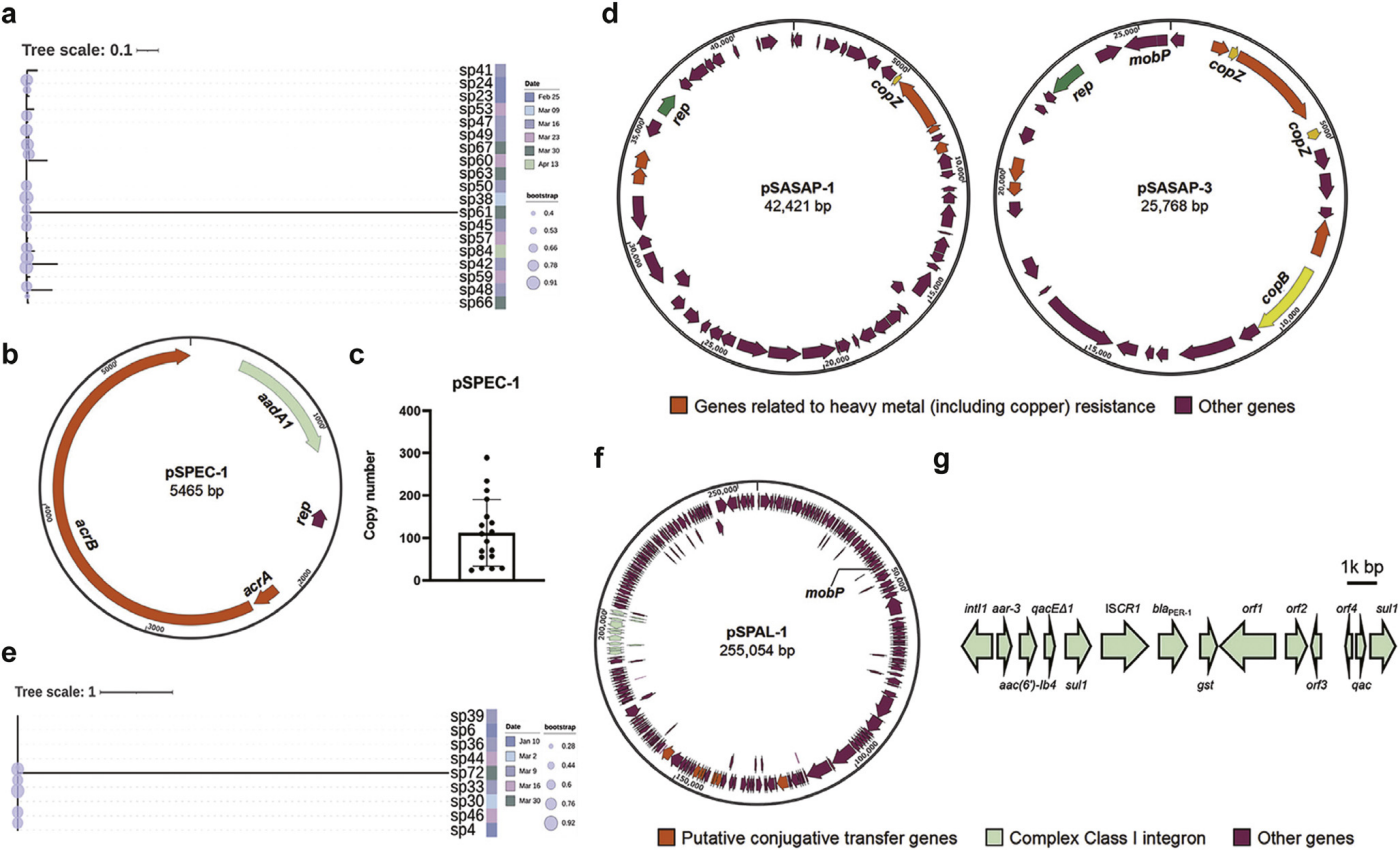
Characterization of the strains and plasmids identified in this study. (a) Phylogenetic analysis of *E. coli*. (b) Schematic map of plasmid pSPEC-1. (c) Copy numbers of pSPEC-1 per cell. (d) Plasmids in *S. saprophyticus*. (e) Phylogenetic analysis if *A. lwoffii* strains. (f) Schematic map of pSAPL-1 identified in *A. lwoffii* strain sp72. (g) Gene organization of the complex Class I integron located on plasmid pSAPL-1.

Nineteen *E. coli*isolates were obtained from swimming pool water between February 25th and April 13th (Table S2). Surprisingly, phylogenetic analysis showed that all *E. coli*isolates, except for *E. coli*isolate sp61, were evolutionarily close (Fig. 4a). On average, these isolates only carry 73.4 mutations in comparison with *E. coli*sp23, much smaller than the 348 mutations found in *E. coli*sp61. Considering these mutations, including single nucleotide polymorphisms, insertion-deletions (InDels), and small replacements, without large structural variations or inversions, these isolates can be considered as the same strain.

Analysis of plasmids in *E. coli*isolates revealed that all *E. coli*isolates, except *E. coli*sp61, carried the same pSPEC-1 plasmid (Fig. 4b). pSPEC-1 belonged to the Col replicon type (Table S5). Sequencing showed that pSPEC-1 carried genes encoding AcrA and AcrB, which are components of the AcrAB-TolC efflux pump. BLAST analysis revealed that the AcrA and AcrB of pSPEC-1 had significant homology with a region of the *E. coli*74D chromosome (CP181454.1), suggesting that this plasmid region might have been acquired from a chromosomal source (Fig. S1a). The encoded AcrA is a C-terminal fragment and AcrB is a variant with a G508P substitution. Furthermore, pSPEC-1 was present at a high copy number in these isolates, with an average of 112 copies per cell (Fig. 4c), suggesting that it may function in the efflux of detrimental chemicals, such as disinfectants.

Fourteen *Staphylococcus* isolates were obtained from swimming pool water (Table S5). Most species of this genus are known pathogenic bacteria that commonly colonize human skin. They can cause various diseases, including skin infections, tissue infections, and bacteremia [36]. Four *S. aureus* isolates were obtained on January 22nd and February 19th. On average, 36 mutations were present between *S. aureus* isolates and the earliest isolated *S. aureus* sp7, suggesting that they are closely related isolates. In addition, they carried the same pSPSA-1 plasmid, which belonged to the rep5a replicon type (Table S5). Taken together, these results suggest that the same *S. aureus* strain persisted for one month under disinfectant pressure, which is in agreement with the observations for *E. coli*. Sequence alignment revealed that pSPSA-1 exhibited 99.69 % identity with an unnamed plasmid from *S. aureus* strain UP_678 (accession number CP047840.1), suggesting that they are highly conserved variants or derivatives of the same plasmid backbone (Fig. S1b).

Similar to *S. aureus*, five *Staphylococcus saprophyticus* isolates were obtained between March 16th and April 13th. The average number of mutations between *S. saprophyticus* isolates and the earliest isolated *S. saprophyticus* sp43 was 25.8. They all contained the same plasmids, pSASAP-1, pSASAP-2, and pSASAP-3 (all belonging to the rep family replicon), confirming that they are evolutionarily close isolates, and further confirming the simplicity of pathogen communities and persistence of the same pathogenic strain under disinfectant stress.

Twenty different plasmids were identified in *Staphylococcus* isolates, including 11 previously reported plasmids and 9 newly identified strains. Two of the three shared plasmids in *S. saprophyticus* isolates, pSASAP-1 and pSASAP-2, were previously observed, as they exhibited high sequence coverage (93 % and 83 %, respectively) and near-complete nucleotide identity (>99.9 %) with known plasmids deposited in public databases (Table S5 and Fig. S1). Comparative analysis revealed that pSASAP-3 shared only 53 % sequence coverage with its closest known plasmid, CP003673.1, suggesting a previously uncharacterized plasmid backbone (Table S5 and Fig. S1e). Further analysis revealed that both pSASAP-1 and pSASAP-3 contained a substantial number of genes involved in heavy metal detoxification, particularly copper (Fig. 4d). The genes *copZ* and *copB* encode copper transporters that effectively reduce the impact of copper on bacteria [37], the remaining proteins encoded by the plasmid also participate in the transport of heavy metals. This may explain the persistence of the same *S. saprophyticus* strain for over a month in the swimming pool, as the carriage of these two plasmids can increase tolerance to CuSO_4_, which was used also as a disinfectant in the pool in this study.

Nine *Acinetobacter lwoffii* isolates were obtained from January 10th to March 30th. Phylogenetic analysis showed that all isolates, except *A. lwoffii* sp72, were phylogenetically close (Fig. 4e), suggesting that they were the same strain. An average of 66.9 mutations were present between the *Acinetobacter* isolates and the earliest isolated *A. lwoffii* sp4 strain. All these *A. lwoffii* isolates carried a large number of plasmids; for instance, *A. lwoffii* sp36 harbored eight plasmids.

The isolation of pathogenic bacterial strains clearly demonstrates that pathogens can survive in a well-operated and disinfected swimming pool. However, only specific strains containing specific genetic determinants can persist under disinfectant challenge, including a novel efflux pump-encoding plasmid in *E. coli*and heavy-metal-resistant plasmids in *S. saprophyticus*. Genetic and phylogenetic analyses of each pathogenic species suggested that only one strain was able to persist across all pathogenic species, *E. coli, S. aureus, S. saprophyticus*, and *A. lwoffii*, whereas the other strains were eliminated. This finding indicates the specificity of the strains capable of surviving in well-disinfected swimming pools.

### 3.4 Identification of a new mobile antibiotic resistant complex class I integron in Acinetobacterlwoffii sp72 from a swimming pool

Antimicrobial susceptibility tests showed that six strains were antimicrobial-resistant (Table S5): *A. lwoffii* sp4 was piperacillin-resistant, *A. lwoffii* sp36 was imipenem-resistant, *E. coli*sp47 was trimethoprim-sulfamethoxazole resistant, *E. coli*sp50 was kanamycin-resistant, *A. lwoffii* sp72 was ceftazidime-resistant, and *S. saprophyticus* sp77 was resistant to both azithromycin and trimethoprim.

Analysis of ARGs led to an interesting finding for *A. lwoffii* sp72: the β-lactam resistant *bla*_PER-1_ gene, which is most likely responsible for its ceftazidime resistance phenotype, is located in a plasmid-harbored complex Class I integron (Fig. 4f). This integron also carries the rifamycin resistance *aar-3* gene and aminoglycoside resistance gene *aac(6′)-Ib4*. In the ISCR1-flanking gene cassette, this integron also carried the glutathione S-transferase-coding gene *gst*, the ABC transporter ATP-binding protein-coding gene *orf1*, a hypothetical protein-coding gene *orf2*, the membrane protein-coding gene *orf3*, and *orf4*, which encodes a putative secreted protein (Fig. 4g). To our knowledge, this is a new complex Class I integron in *A. lwoffii*, given the limited number of reports on such elements in this nosocomial pathogen. In addition, it is intriguing to find that this integron is located on the pSPAL-1 plasmid. pSPAL-1 harbored an uncharacterized replication system and showed high overall homology with plasmid CP042547.1, despite the presence of gene rearrangement regions (Table S5). Notably, their complex Class I integron regions were not fully aligned, suggesting structural variation and likely independent evolution of the integron-associated accessory region (Fig. S1f). Genetic analysis further revealed that pSPAL-1 carried a MOBP-type relaxase and putative conjugation-related genes (Fig. 4f). Therefore, this integron has significant mobility potential, as it can be transferred with a conjugative plasmid, which may also aggravate the threat of antimicrobial resistance.

## 4 Discussion

Artificial swimming pools are open environments to which human activities continually introduce diverse bacteria. Water disinfection, encompassing both biological and industrial methods, is essential for public health [38,39]. Therefore, industrial disinfection serves as the primary intervention strategy for microbial control and maintenance of hygienic water quality in swimming pools. Although some studies have characterized the microbial composition of swimming pool ecosystems [13,40], the temporal dynamics and changes in these microbial communities remain largely unexplored. To our knowledge, this is the first systematic investigation of microbial dynamics before and after the opening of a new public swimming pool.

Continuous sampling and bacterial composition analysis revealed that Proteobacteria was the predominant phylum across all samples; it maintained its predominance even under disinfection pressure. This finding aligns with previous reports on the ecological dominance of Proteobacteria in aquatic environments [41,42]. The persistence of this phylum under disinfectant stress may be attributed to two key adaptive mechanisms: (1) enhanced biofilm formation capacity, which provides inherent protection against environmental stressors such as disinfectants [38], and (2) chlorine resistance through nitrification processes [43]. This ecological advantage under disinfectant stress has been well-documented, with Proteobacteria demonstrating selective survival in chlorinated environments [44].

Genus-level bacterial composition analysis revealed distinct shifts in the microbial communities before and after pool operation. The pre-operation samples were dominated by *Escherichia-Shigella, Pseudomonas, Acinetobacter*, and *Hyphomicrobium*, which are bacteria associated with humans. Considering that the initial water was from an urban water supply system, this composition reflects the microbial community in tap water, which is related to human activities/contamination and wastewater treatment [45]. The post-operation samples from the swimming pool water were dominated by four groups of environmental bacteria. Hyphomonadaceae and *Porphyrobacter* are marine bacteria [22,24], while *Rhizobiaceae* and *Nevskia* are soil bacteria [27,29]. The swimming pool analyzed in this work is located on a campus that is only 500 m from the Yellow Sea of China, where marine bacteria originate and soil bacteria can be carried by swimmers. It can be observed from these bacterial groups that human inoculation is a major determinant of the microbial community structure in swimming pools.

We discovered a gene encoding the AcrAB subunits in the plasmid pSPEC-1 in *E. coli.* AcrAB-TolC is a non-specific efflux pump complex found in Enterobacteriaceae that pumps antibiotics and other detrimental chemicals [46]. Although plasmid-borne RND pumps, such as *tmexCD-oprJ,* have been observed in Enterobacteriaceae such as *Klebsiella pneumoniae* [47], AcrAB-TolC remains exclusively chromosomal in *E. coli*. The acquisition of efflux pumps via MGEs represents a key adaptive strategy for rapid environmental adaptation along with other mechanisms, such as enzyme-mediated detoxification [48]. It needs to be emphasized here that this novel ‘pump plasmid’ may cause collateral consequences, as the AcrAB-TolC complex is known to confer resistance to a broad spectrum of antibiotics, including β-lactams, azithromycin, trimethoprim, quinolones, and the last-line antibiotic tigecycline [49,50]. However, in the present study, none of the *E. coli*strains harboring AcrAB exhibited significant drug resistance (Table S5). The AcrAB-TolC system alone does not confer high-level resistance, because its effectiveness depends on synergistic interactions with other resistance mechanisms [51]. In contrast, the truncation of AcrA and mutations in AcrB may significantly affect drug resistance.

Phylogenetic analysis of the isolated bacteria revealed that only identical strains persisted under continuous disinfectant pressure. Notably, even for highly prevalent species, such as *E. coli*and *S. aureus*, no strain diversity was observed in the surviving populations. However, two transient isolates (*E. coli*sp61 and *A. lwoffii* sp72) with distinct genomic architectures were uniquely detected during the March 30 sampling (subsequent samples were culture-negative). We hypothesized that the transient detection of *E. coli*sp61 and *A. lwoffii* sp72 in this sampling time may be attributed to the sustained high swimmer load prior to sampling, which likely increased the microbial inputs from swimmers. No *A. lwoffii* strains (including sp72) were detected in the subsequent analyses, confirming that *A. lwoffii* sp72 is a transient human-introduced strain. In contrast, although later samples remained positive for *E. coli*, the specific strain sp61 was lost. Given that pool disinfectants exert persistent selective pressure [52], we propose that the absence of *E. coli* sp61 resulted from a lack of specialized genetic determinants (efflux pumps or copper resistance-conferring plasmids), rendering it noncompetitive under these conditions. Because bacteria with specific genetic determinants are continuously enriched through reproduction under selective pressure, they eventually occupy the ecological niches of *E. coli* sp61, leading to its failure to be detected in subsequent sample tests. This ecological succession pattern aligns with the classic “selection-sieving” model observed in disinfectant-stressed aquatic environments [44,52], the composition and abundance of bacteria change with the use of disinfectants, which is the same as the results of our study.

This study represents the first comprehensive time-series analysis of microbial community dynamics in swimming pools, revealing the critical role of disinfectants in shaping the microbial structure. Our findings demonstrate that reduced disinfectant stress rapidly restores community complexity and facilitates microbial rebalancing, thereby highlighting the resilience of the system. Our findings also describes how metabolically specialized, uncommon microbes persist in this unique ecosystem. However, this study had several limitations that highlight future research directions. The main limitation of our study stems from its reliance on bacterial culture and 16S rRNA gene sequencing. This approach restricted our ability to fully characterize anaerobic and unculturable microorganisms and perform detailed genetic analyses, particularly regarding the numerous plasmids we observed, whose ecological significance remains to be fully elucidated. Consequently, a powerful strategy for future research in this area would be to employ shotgun metagenomics to reconstruct metagenome-assembled genomes (MAGs). This would not only provide a more comprehensive taxonomic and functional profile of uncultured taxa but also enable direct plasmid characterization. Similarly, our findings generated specific hypotheses regarding disinfectant tolerance mechanisms that require direct experimental validation. Unlike the broad ecological insights from MAGs, a paramount future goal is to perform targeted phenotypic assays to confirm the functions of the identified genetic elements in the isolated strains. This orthogonal approach is crucial to establish causative links beyond genomic correlations.

## 5 Conclusions

**Fig. 5:**
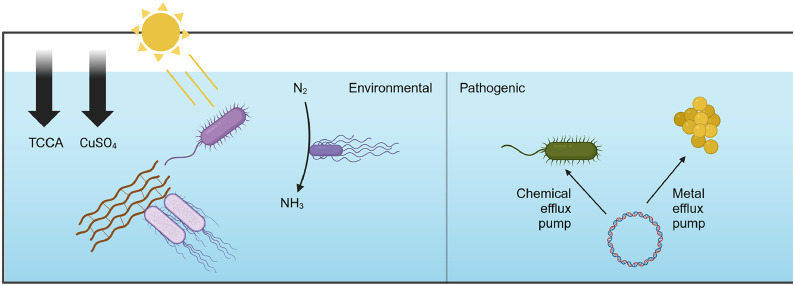
Persistence of specialized bacteria in swimming pools.

The bacterial content of swimming pool water was studied in a newly opened swimming pool by following the bacterial community structures and genomics of the isolated bacteria through time. The disinfection process has a significant impact on the bacteria in pool water by nearly eliminating pathogenic bacteria and drastically and temporarily simplifying the bacterial community. It was found that specialized bacteria can persist in swimming pools despite very strong disinfectant stress (Fig. 5); metabolically specialized environmental bacteria can persist under nutrient- and disinfectant-stressed conditions, and genetically specialized pathogenic bacteria can persist due to the acquisition of unique plasmids. A new complex Class I integron, which may have antibiotic consequences, was also found in a pathogenic bacterium. This underscores its potential public health risk, as these elements can serve as reservoirs for the capture and dissemination of ARGs. This study describes the microbial community in swimming pool water, a common yet rarely studied public water body, and provides insights into the survival of environmental and pathogenic bacteria in this harsh environment. Our study demonstrated that a management strategy based solely on disinfection is insufficient; it must be supplemented with new protocols to specifically monitor and address persistent bacterial and ARGs in water.

## Data availability statement

The sequencing data generated in this study are available in the GenBank database under accession number PRJNA1211488.

## CRediT authorship contribution statement

**Ziyu Wan:** Investigation, Formal analysis. **Wanzhen Guo:** Software, Investigation. **Kaiyue Zhang:** Investigation. **Lianfeng Tang:** Conceptualization. **Youming Zhang:** Supervision. **Hai Xu:** Supervision. **Mengge Zhang:** Methodology. **Xueyun Geng:** Methodology, Funding acquisition. **Ling Li:** Funding acquisition. **Wenjia Wang:** Writing – review & editing, Methodology. **Mingyu Wang:** Writing – original draft, Funding acquisition.

## Declaration of competing interest

The authors declare the following financial interests/personal relationships which may be considered as potential competing interests: Given his role as Editor-in-Chief, Dr. Youming Zhang had no involvement in the peer-review of this article and has no access to information regarding its peer-review. Full responsibility for the editorial process for this article was delegated to Dr. Biao Tang. Mingyu Wang reports financial support was provided by National Key Research and Development Program of China. Mingyu Wang reports financial support was provided by Qingdao Science and Technology Wellness Promotion Demonstration Program. Mingyu Wang reports financial support was provided by National Natural Science Foundation of China. Mingyu Wang reports financial support was provided by SKLMT Frontiers and Challenges Project. Ling Li reports financial support was provided by Shandong Provincial Natural Science Foundation. Ling Li reports financial support was provided by Qingdao Natural Science Foundation. Xueyun Geng reports financial support was provided by Opening Project of Shanghai Key Laboratory of Atmospheric Particle Pollution and Prevention (LAP). If there are other authors, they declare that they have no known competing financial interests or personal relationships that could have appeared to influence the work reported in this paper.

## Appendix Supplementary materials

Image, image 1 dummy alt text

Image, application 2

Image, application 3

Image, application 4

Image, application 5

Image, application 6

Image, application 7

Image, application 8

Image, application 9
