# Supplementary figures and images for: Persistence of specialized bacteria during disinfectant challenge in a new swimming pool
Source: Eng Microbiol. 2026 Feb 5;6(2):100261. doi: 10.1016/j.engmic.2026.100261 (PMC13323885; doi:10.1016/j.engmic.2026.100261)

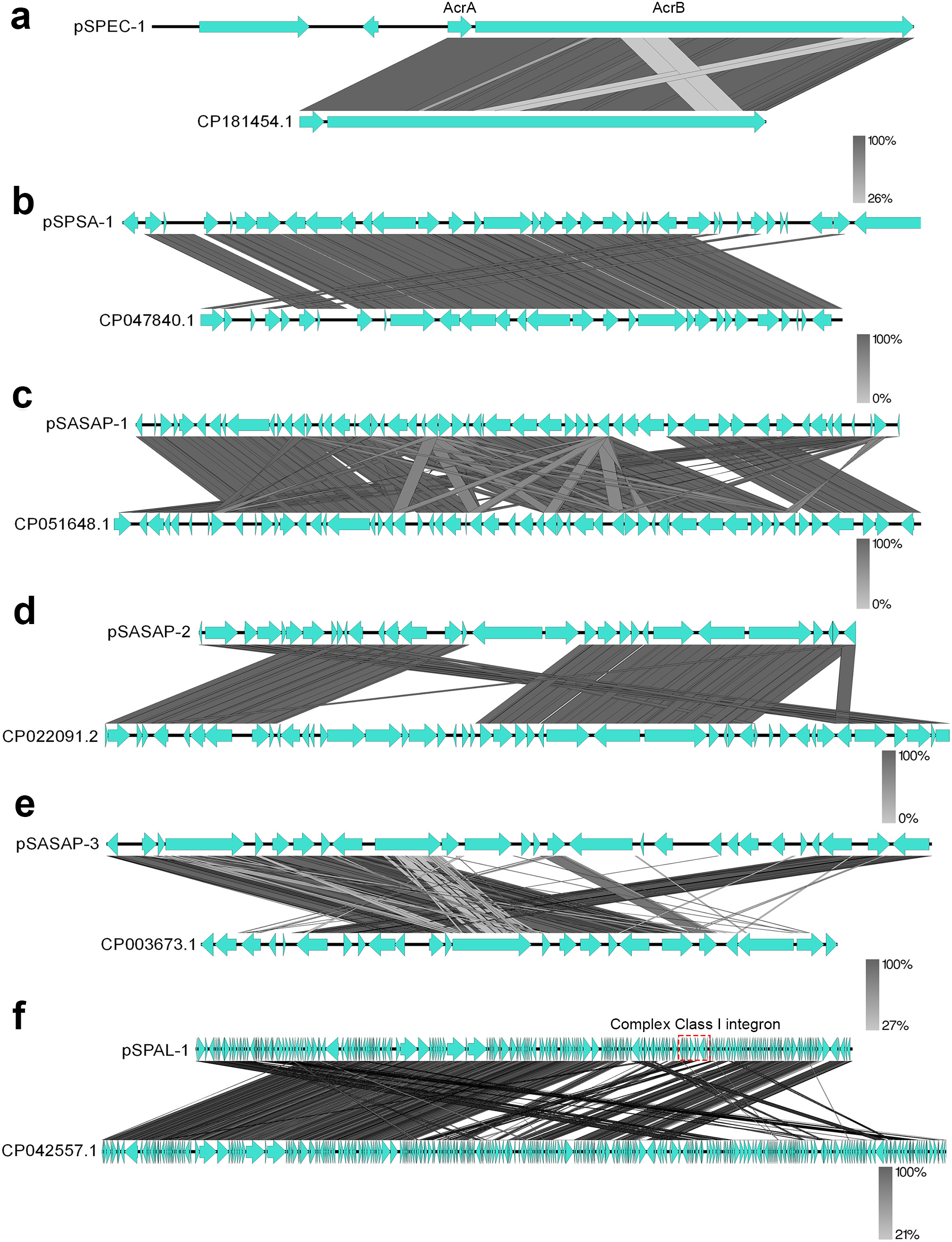

Supplement: Supplementary file 1 [file mmc1.jpg]
