# Supplementary material for: Persistence of specialized bacteria during disinfectant challenge in a new swimming pool
Source: Eng Microbiol. 2026 Feb 5;6(2):100261. doi: 10.1016/j.engmic.2026.100261 (PMC13323885; doi:10.1016/j.engmic.2026.100261)
Supplement: Supplementary file 2 [file mmc2.docx]

**Table S1. Bacterial strains isolated in this work.**

| **Strain** | **Collection date** | **Accession number** |
| --- | --- | --- |
| *Bacillus cereus* sp1 | 2024.01.10 | JBMQAJ000000000 |
| *Bacillus cereus* sp3 | 2024.01.10 | JBMQAI000000000 |
| *Acinetobacter lwoffii* sp4 | 2024.01.10 | JBMQAH000000000 |
| *Bacillus cereus* sp5 | 2024.01.10 | JBMQAG000000000 |
| *Acinetobacter lwoffii* sp6 | 2024.01.10 | JBMQAF000000000 |
| *Staphylococcus aureus* sp7 | 2024.01.22 | JBMQAE000000000 |
| *Bacillus velezensis* sp8 | 2024.01.22 | JBMQAD000000000 |
| *Bacillus licheniformis* sp9 | 2024.01.22 | JBMQAC000000000 |
| *Bacillus velezensis* sp10 | 2024.01.22 | JBMQAB000000000 |
| *Staphylococcus aureus* sp11 | 2024.01.29 | JBMQAA000000000 |
| *Staphylococcus epidermidis* sp12 | 2024.01.29 | JBMPZZ000000000 |
| *Bacillus velezensis* sp13 | 2024.01.29 | CP186483 |
| *Bacillus cereus* sp14 | 2024.01.29 | JBMPZY000000000 |
| *Staphylococcus aureus* sp15 | 2024.01.29 | JBMPZX000000000 |
| *Bacillus cereus* sp16 | 2024.02.19 | JBMPZW000000000 |
| *Staphylococcus hominis* sp17 | 2024.02.19 | JBMPZV000000000 |
| *Bacillus safensis* sp18 | 2024.02.19 | CP186482 |
| *Neobacillus sp005154805* sp19 | 2024.02.19 | JBMPZU000000000 |
| *Bacillus cereus* sp20 | 2024.02.19 | JBMPZT000000000 |
| *Staphylococcus aureus* sp21 | 2024.02.19 | JBMPZS000000000 |
| *Bacillus thuringiensis* sp22 | 2024.02.25 | JBMPZR000000000 |
| *Escherichia coli* sp23 | 2024.02.25 | JBMPZQ000000000 |
| *Escherichia coli* sp24 | 2024.02.25 | JBMPZP000000000 |
| *Bacillus cereus* sp25 | 2024.03.02 | JBMPZO000000000 |
| *Bacillus velezensis* sp26 | 2024.03.02 | JBMPZN000000000 |
| *Bacillus cereus* sp27 | 2024.03.02 | JBMPZM000000000 |
| *Bacillus cereus* sp28 | 2024.03.02 | JBMPZL000000000 |
| *Bacillus thuringiensis* sp29 | 2024.03.02 | JBMPZK000000000 |
| *Acinetobacter lwoffii* sp30 | 2024.03.02 | JBMPZJ000000000 |
| *Staphylococcus haemolyticus* sp31 | 2024.03.02 | JBMPZI000000000 |
| *Kocuria palustris* sp32 | 2024.03.02 | CP186481 |
| *Acinetobacter lwoffii* sp33 | 2024.03.09 | JBMPZH000000000 |
| *Bacillus velezensis* sp34 | 2024.03.09 | JBMPZG000000000 |
| *Bacillus cereus* sp35 | 2024.03.09 | JBMPZF000000000 |
| *Acinetobacter lwoffii* sp36 | 2024.03.09 | JBMPZE000000000 |
| *Bacillus subtilis* sp37 | 2024.03.09 | JBMPZD000000000 |
| *Escherichia coli* sp38 | 2024.03.09 | JBMPZC000000000 |
| *Acinetobacter lwoffii* sp39 | 2024.03.09 | JBMPZB000000000 |
| *Priestia zanthoxyli* sp40 | 2024.03.09 | JBMPZA000000000 |
| *Escherichia coli* sp41 | 2024.03.16 | JBMPYZ000000000 |
| *Escherichia coli* sp42 | 2024.03.16 | JBMPYY000000000 |
| *Staphylococcus saprophyticus* sp43 | 2024.03.16 | JBMPYX000000000 |
| *Acinetobacter lwoffii* sp44 | 2024.03.16 | JBMPYW000000000 |
| *Escherichia coli* sp45 | 2024.03.16 | JBMPYV000000000 |
| *Acinetobacter lwoffii* sp46 | 2024.03.16 | JBMPYU000000000 |
| *Escherichia coli* sp47 | 2024.03.16 | JBMPYT000000000 |
| *Escherichia coli* sp48 | 2024.03.16 | JBMPYS000000000 |
| *Escherichia coli* sp49 | 2024.03.16 | JBMPYR000000000 |
| *Escherichia coli* sp50 | 2024.03.16 | JBMPYQ000000000 |
| *Bacillus velezensis* sp51 | 2024.03.23 | JBMPYP000000000 |
| *Staphylococcus haemolyticus* sp52 | 2024.03.23 | JBMPYO000000000 |
| *Escherichia coli* sp53 | 2024.03.23 | JBMPYN000000000 |
| *Staphylococcus lugdunensis* sp54 | 2024.03.23 | JBMPYM000000000 |
| *Bacillus velezensis* sp55 | 2024.03.23 | JBMPYL000000000 |
| *Escherichia coli* sp57 | 2024.03.23 | JBMPYK000000000 |
| *Bacillus velezensis* sp58 | 2024.03.23 | JBMPYJ000000000 |
| *Escherichia coli* sp59 | 2024.03.23 | JBMPYI000000000 |
| *Escherichia coli* sp60 | 2024.03.23 | JBMPYH000000000 |
| *Escherichia coli* sp61 | 2024.03.30 | JBMPYG000000000 |
| *Bacillus thuringiensis* sp62 | 2024.03.30 | JBMPYF000000000 |
| *Escherichia coli* sp63 | 2024.03.30 | JBMPYE000000000 |
| *Bacillus velezensis* sp64 | 2024.03.30 | JBMPYD000000000 |
| *Bacillus velezensis* sp65 | 2024.03.30 | JBMPYC000000000 |
| *Escherichia coli* sp66 | 2024.03.30 | JBMPYB000000000 |
| *Escherichia coli* sp67 | 2024.03.30 | JBMPYA000000000 |
| *Bacillus velezensis* sp68 | 2024.03.30 | JBMPXZ000000000 |
| *Bacillus velezensis* sp70 | 2024.03.30 | JBMPXY000000000 |
| *Bacillus licheniformis* sp71 | 2024.03.30 | JBMPXX000000000 |
| *Acinetobacter lwoffii* sp72 | 2024.03.30 | JBMPXW000000000 |
| *Bacillus velezensis* sp73 | 2024.03.30 | JBMPXV000000000 |
| *Bacillus thuringiensis* sp74 | 2024.03.30 | JBMPXU000000000 |
| *Bacillus velezensis* sp75 | 2024.03.30 | JBMPXT000000000 |
| *Bacillus cereus* sp76 | 2024.04.06 | JBMPXS000000000 |
| *Staphylococcus saprophyticus* sp77 | 2024.04.06 | JBMPXR000000000 |
| *Staphylococcus saprophyticus* sp78 | 2024.04.06 | JBMPXQ000000000 |
| *Bacillus cereus* sp79 | 2024.04.06 | JBMPXP000000000 |
| *Bacillus velezensis* sp80 | 2024.04.06 | JBMPXO000000000 |
| *Bacillus cereus* sp81 | 2024.04.06 | JBMPXN000000000 |
| *Bacillus cereus* sp82 | 2024.04.06 | JBMPXM000000000 |
| *Staphylococcus saprophyticus* sp83 | 2024.04.06 | JBMPXL000000000 |
| *Escherichia coli* sp84 | 2024.04.13 | JBMPXK000000000 |
| *Staphylococcus saprophyticus* sp85 | 2024.04.13 | JBMPXJ000000000 |
| *Bacillus cereus* sp87 | 2024.04.13 | JBMPXI000000000 |
